# Privacy-Preserving Deep Speaker Separation for Smartphone-Based Passive Speech Assessment

**DOI:** 10.1109/OJEMB.2021.3063994

**Published:** 2021-03-04

**Authors:** Apiwat Ditthapron, Emmanuel O. Agu, Adam C. Lammert

**Affiliations:** Computer Science DepartmentWorcester Polytechnic Institute 8718 Worcester MA 01609 USA; Biomedical Engineering DepartmentWorcester Polytechnic Institute 8718 Worcester MA 01609 USA

**Keywords:** Mel-Frequency Cepstrum Coefficients (MFCCs), overlapped speech, speaker representation, speech separation, Impact Statement—The proposed Deep-MASKS mitigates cross-talk in speech encoded as MFCC features, which are widely utilized to preserve voice privacy in passive health assessment and other speech applications on smartphones

## Abstract

*Goal:* Smartphones can be used to passively assess and monitor patients’ speech impairments caused by ailments such as Parkinson’s disease, Traumatic Brain Injury (TBI), Post-Traumatic Stress Disorder (PTSD) and neurodegenerative diseases such as Alzheimer’s disease and dementia. However, passive audio recordings in natural settings often capture the speech of non-target speakers (cross-talk). Consequently, speaker separation, which identifies the target speakers’ speech in audio recordings with two or more speakers’ voices, is a crucial pre-processing step in such scenarios. Prior speech separation methods analyzed raw audio. However, in order to preserve speaker privacy, passively recorded smartphone audio and machine learning-based speech assessment are often performed on derived speech features such as Mel-Frequency Cepstral Coefficients (MFCCs). In this paper, we propose a novel Deep MFCC bAsed SpeaKer Separation (Deep-MASKS). *Methods:* Deep-MASKS uses an autoencoder to reconstruct MFCC components of an individual’s speech from an i-vector, x-vector or d-vector representation of their speech learned during the enrollment period. Deep-MASKS utilizes a Deep Neural Network (DNN) for MFCC signal reconstructions, which yields a more accurate, higher-order function compared to prior work that utilized a mask. Unlike prior work that operates on utterances, Deep-MASKS operates on continuous audio recordings. *Results:* Deep-MASKS outperforms baselines, reducing the Mean Squared Error (MSE) of MFCC reconstruction by up to 44% and the number of additional bits required to represent clean speech entropy by 36%.

## I. Introduction

Growing evidence suggests that frequent monitoring of speech can help clinicians diagnose and steer the course of treatment for speech disorders at an early stage [1], [2]. Moreover, various neurological disorders such as Parkinson’s disease and Traumatic Brain Injury (TBI) manifest in patients’ speech, making speech-based assessments an exciting, emerging research area [2]–[5]. Current research utilizes smartphones to remotely monitor patients more frequently due to their near-ubiquitous ownership and powerful processing capabilities, and advancements in speech processing techniques. Consequently, cost-effective, passive, clinically valid voice assessments are now feasible using smartphones [5]–[7]. Smartphones facilitate remote assessment of patients who cannot visit the clinic regularly. Such assessments can be done either in real-time or offline by analyzing recorded speech. Passive, continuous speech assessments in natural settings provide comprehensive insights into patients’ health and lifestyles outside the clinic, augmenting traditional screening performed by clinicians. Prior work has demonstrated promising results for smartphone-based voice screenings to detect speech impairments caused by Parkinson’s disease [8], and neurodegenerative diseases such as Alzheimer’s disease and dementia [9], [10], neurocognitive deficits, Traumatic Brain Injury (TBI) and Post-Traumatic Stress Disorder [11].

**Fig. 1.**
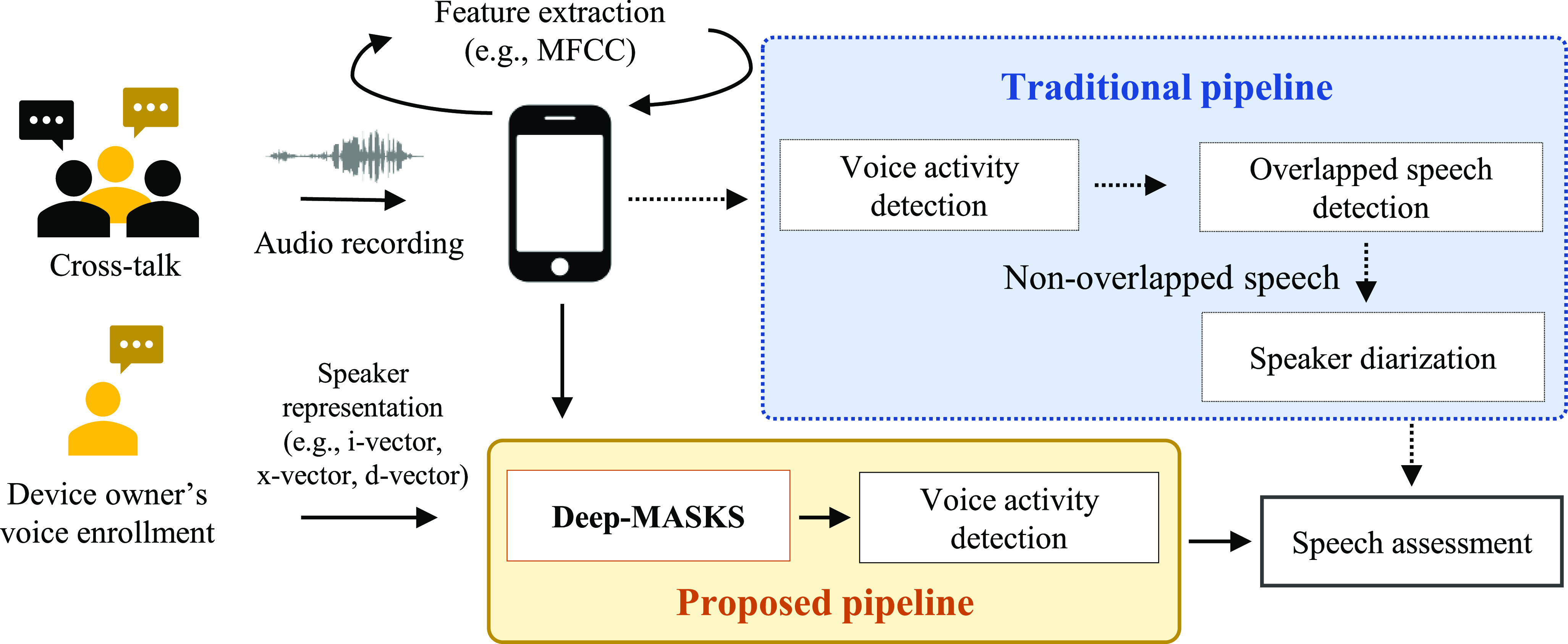
A comparison between our proposed pipeline and the traditional processing pipeline for passive smartphone-based speech assessment. The smartphone simultaneously records audio and extracts speech features, which reduces data storage requirements and preserves speaker privacy. In the traditional pipeline, the processing steps preceding speech assessment include Voice Activity Detection (VAD), which is used for locating speech utterances, followed by overlapped speech detection, and speaker diarization (detecting turn-taking in speech). We introduce an alternate Deep-MASKS pipeline which performs the pre-assessment step of removing the speech of non-target speakers that is then followed by VAD.

Fig. 1 shows a passive speech assessment pipeline that is typically utilized for smartphones, distinguishing the traditional speech processing pipeline in comparison to our novel Deep-MASKS pipeline. For instance, Huang *et al.* [12] utilized a similar traditional speech pipeline to detect depressed speech, Their pipeline utilized Support Vector Machines (SVMs) to classify audio in the form of Mel-Frequency Cepstral Coefficients (MFCCs) features, which represent speech as the Discrete Cosine Transform (DCT) of the log power frequency spectrum on the Mel frequency scale – detailed computational steps are provided in the supplementary materials. MFCCs have been widely adopted in health-oriented speech technologies, and in many modern speech technologies [13]–[15]. The MFCC representation of speech is also attractive in smartphone-based speech analytics because of its compact size and the fact that it exhibits less correlations than filter bank features [16]. Consequently, MFCC features are often utilized in most steps of a typical smartphone voice analytics pipeline and related tasks, including automatic speech recognition, speaker diarization, and speech assessment, as they have been found to outperform low-level features such as fundamental frequency and loudness, zero-crossing rate [13], [14]. In order to preserve the speaker’s privacy in passive health assessments, which is important in healthcare, audio is frequently featurized as MFCCs feature on the source smartphone and utilized in subsequent speech processing steps. Additionally, MFCC-based encryption algorithms for remote healthcare systems have been proposed to make them more secure [17], [18]. One known drawback of of MFCCs is that they compromise the richness of the speaker’s voice.

*The problem:* MFCC features are not robust to background noise and cross-talk (overlapped speech). Cross-talk, which occurs when a smartphone captures background speech that does not belong to its owner, is a common problem when smartphones are used for passive assessment in natural environments. Robust solutions for cross-talk are crucial to avoid erroneously assessing the speech of a non-target speaker. Cross-talk has been found [19], [20] to be one of the factors that cause major degradation factors in speaker diarization. They proposed introducing the detection of overlapped speech into the traditional speech pipeline as illustrated in Fig. 1. If detected, overlapped speech can be excluded prior to speech assessment. While discarding overlapped speech improves speaker diarization performance, it may not be appropriate for applications with only limited data. In this paper, we present a novel method to solve the cross-talk problem.

*Background and prior work:* Various speaker separation strategies have previously been proposed to isolate the speech of a specific speaker [21]–[23]. Speech produced by a speaker is widely represented using speaker representations, such as i-vector [24], d-vector [25] and x-vector [26], which map individual voice characteristics to a fixed-size vector in a high dimensional feature-space. These speaker representations were originally developed for the speaker recognition task and have previously been utilized in speech separation [22], [23]. In their VoiceFilter system, Wang *et al.* [22] used the d-vector representation to isolate target speech, utilizing a mask generated using deep learning. Corresponding speech components contained in a spectrogram were then filtered before transformation back to raw audio. SpeakerBeam [23] used the i-vector representation as one of their auxiliary speaker inputs to neural networks with an architecture similar to that of Wang et al’s VoiceFilter. [22].

Speaker separation methods, which target scenarios in which no prior information on the speaker, their identity or representations of their speech are required, have also been proposed. Luo *et al.* [21] introduced deep attractor networks to separate the speaker using an attractor point in speaker embedding-space deep clustering. Nachamani *et al.* [27] proposed a multiple-step model for situations in which the number of speakers is unknown, and trained the model separately for all possible numbers of speakers. Although these methods do not require knowing the identity of the speaker, they require additional pre-processing or post-processing step(s) to identify the number of speakers within an audio segment. On smartphones, which typically have one user and on which computational resources are often limited, generating a representation of the speaker’s voice once in order to obviate the need to perform the speaker identification step many times, is a good trade-off.

*Our approach:* Most prior speaker separation approaches focused on analyzing the audio waveform. In contrast, we propose a Deep MFCC bAsed SpeaKer Separation (Deep-MASKS) network that separates speech in feature space using MFCCs. Applying Deep-MASKS on MFCCs as a data pre-processing step enables complete integration into the typical MFCC-based speech assessment pipeline shown in Fig. 1, and is able to utilize any of the most popular speaker representations. Deep-MASKS makes novel use of a deep learning-based autoencoder and speaker representation vector to reconstruct MFCC components that correspond to the target speaker. The method isolates the MFCCs produced by a registered user (smartphone’s owner) while rejecting MFCC features produced by other speakers in an overlapped speech segment. Including a robust speaker separation step using Deep-MASKS ensures that the speech assessed for impariments truly belongs to the owner of the phone, increasing confidence in results produced.

While Deep-MASKS shares some similarities with Voicefilter [22], our method is explicitly designed for frame-level features – as opposed to the raw speech waveform – that are now widely used in many recent speech processing and assessment systems. Deep-MASKS can also flexibly utilize most speaker representation (or embedding) methods and reconstructs MFCCs from a long recording that are more relevant to continuous passive audio assessments rather than utterances, as in VoiceFilter [22]. Deep-MASKS also improves on VoiceFilter by using a DNN for MFCC signal reconstruction instead of a mask, which yields a more accurate higher order reconstruction function. This study evaluates Deep-MASKS on how much the reconstructed MFCCs deviate from MFCCs generated from clean speech using Mean Square Error(MSE), Kullback-Leibler divergence, and Jensen-Shannon divergence error metrics. Our method reduces cross talk from other speakers by up to 44% in overlapped speech. We also include two additional evaluations in which Deep-MASKS is employed as a pre-processing step for Automatic Speaker Recognition (ASR) and speaker diarization tasks in the supplementary materials

*Challenges:* Two main challenges presented by speech separation methods that utilize speaker representations are 1) Insufficient information about speakers and 2) Speaker representations are often restricted to a specific system. We address the limited speaker information issue by considering only one speaker (the smartphone owner) as a target when extracting speech components from the mixture at a time. Threshold values that separate target and non-target speakers are learned using the encoder, speaker matching, and MFCC refinement networks. Our method is also not restricted to a specific system as the speaker matching network is able to adapt to all three previously proposed speaker representations (i-vector, d-vector and x-vector).

## II. Materials and Methods

### A. Speaker Representations

A speaker representation is a high-dimensional vector that represents target speaker information, extracted from multiple utterances in a task-independent fashion. It is included in most speaker-independent tasks in order to cope with the speaker adaptation problem [15], [28]. Saon *et al.* [28] demonstrated that incorporating the i-vector speaker representation with MFCCs improves ASR performance, obviating the need for additional methods that personalize feature values to speakers such as Vocal Tract Length Normalization (VTLN) and feature space maximum likelihood linear regression. In a similar spirit, we employed the *i-vector*, *x-vector* and *d-vector* speaker representation vectors, in the DNN to separate speech that belongs to a smartphone owner.

The *i-vector* is based on a Universal Background Model (UBM), which is a Gaussian Mixture Model (GMM) trained on a multi-speaker corpus. It uses factor analysis to compute low-dimensional total variability factors ($w$) from the super-vector ($M$) as shown in 1, where $m$ is a speaker-independent super-vector from UBM; $T$ is a total-variability matrix. The i-vector is obtained from $w$ and is the only speaker representation method that is not a DNN-based speaker representation, but it has previously been adopted in DNNs to adapt features learned from multiple speakers [15], [28].

\begin{equation*}
M = m + Tw \tag{1}
\end{equation*}

Due to its impressive performance in ASR systems [15], [28], the *d-vector* utilizes Deep Neural Networks (DNNs) to generate an embedding of the speaker for the speaker verification task. The d-vector was initially developed for text-dependent [29] and text-independent speaker verification [25] using Convolutional Neural Networks (CNNs) and Fully-Connected (FC) layers. The main advantage of the d-vector is that it requires fewer utterance frames than the i-vector [25]. However, its embedding vector does not follow a normal distribution like $w$ in the i-vector. *X-vector* utilizes Probabilistic Linear Discriminant Analysis (PLDA), a generative probability model that assumes that the input is a known distribution, to extend the d-vector in a way that preserves a normal distribution. Similar to d-vector, x-vector uses a time-delay neural network to build a speaker representation (feature extraction) with classification done using a PLDA classifier. In speaker recognition, X-vector often outperforms i-vector for short utterances while having comparable performance for long utterances [26].

### B. Deep-Masks

We propose Deep-MASKS, a neural network that reconstructs MFCC features corresponding to a (*target speaker*) using an AutoEncoder (AE) and the i-vector, x-vector, d-vector or any fixed-length speaker representation computed from clean speech gathered during an enrollment period.

**Fig. 2.**
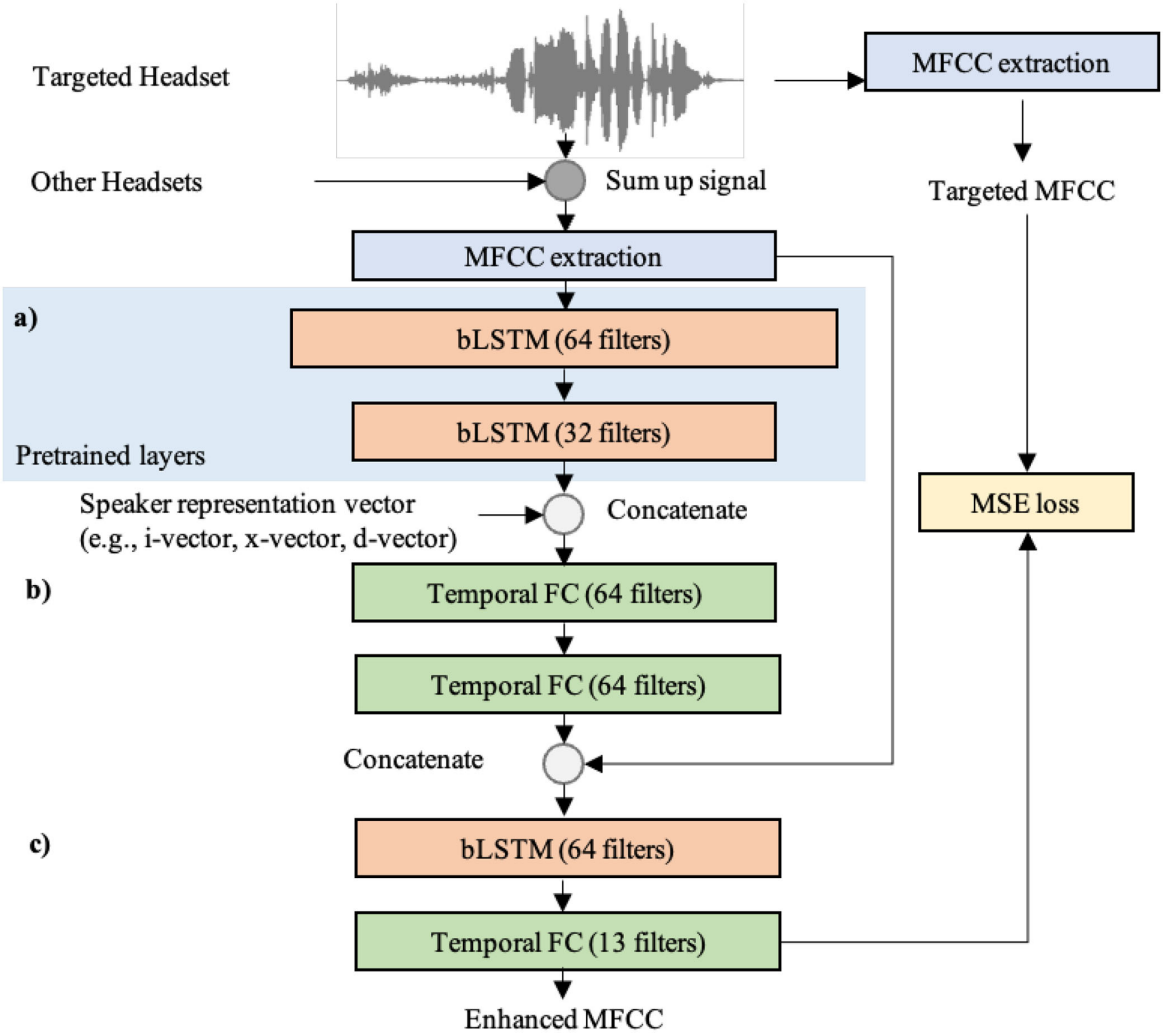
Architecture of the Deep-MASKS using AE with speaker representation, comprising (a) encoder network, (b) speaker matching network, and (c) MFCC refinement network.

**Fig. 3.**
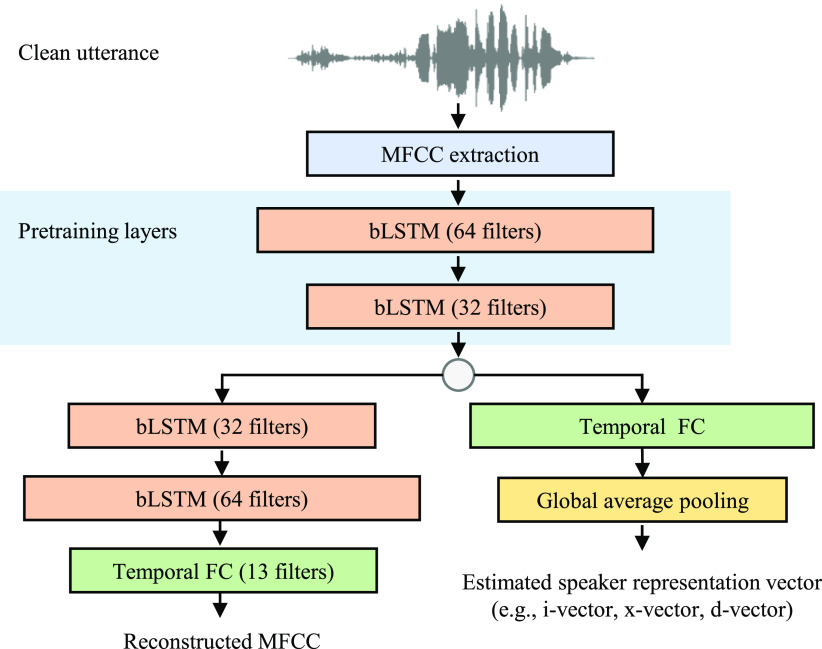
AE used for pre-training encoder network.

Deep-MASKS contains three neural networks: 1) encoder network, 2) speaker matching network and 3) MFCC refinement network as shown in Fig. 2. The encoder network follows the AE concept by transforming an MFCC input into a compact bottleneck feature that maintains speaker information, constrained by an objective function to extract speaker representation during pre-training. Deep-MASKS recognizes a target speaker based on a given speaker representation vector concatenated to the bottleneck feature as an input to the speaker matching network, which applies non-linear filters to remove non-target speakers from the bottleneck representation. In the final stage, the MFCC refinement network reconstructs MFCCs of the target speaker from the bottleneck feature with access to the MFCC input to recover information that might be lost during in the encoder network. The encoder network is pre-trained to reconstruct the MFCC signal and extracts the speaker representation simultaneously, as illustrated in Fig. 3. The parameters of the encoder network ($f_{\theta _1}$) are pre-trained to reconstruct a clean utterance ($y_{ij}$), where $i$ denotes the MFCC frame and $j$ denotes the MFCC coefficient, using a decoder ($h()$) as shown in 2. The encoder network also learns to extract speaker representation vector ($s_{ij}$) from a clean utterance using shared parameters ($f_{\theta _1}$) and a network $g()$, which contains a global average pooling layer to summarize a speaker representation vector by averaging multiple speaker representations over frames.

\begin{align*}
 L_{pretrain} =& \alpha \sum _{i=0}^{n}\sum _{j=0}^{13}{(y_{ij}-h(f_{\theta _1}(y_{ij})))^2} \\
& +(1-\alpha )\sum _{i=0}^{n}\sum _{j=0}^{13}{(s_{ij}-g(f_{\theta _1}(y_{ij})))^2} \tag{2}
\end{align*}

The encoder network is trained to reduce the loss, $L_{pretrain}$ that is a weighted combination of the MFCC reconstruction and L2 norm errors between the predicted speaker representation vector ($g(f_{\theta _1}()$) and the actual speaker representation generated from clean speech gathered during speaker enrollment. The weight ($\alpha$) is set to 20%, which we found optimal for adapting the networks to match speaker representation vectors from other systems. This pre-training allows the whole model to learn parameters of speaker matching ($f_{\theta _2}$) and MFCC refinement ($f_{\theta _3}$), which occurs deeper in the neural network rather than adapting to the speaker representation vector.

During pre-training, a bottleneck is created to encode the speaker representation from the input MFCC. Two layers of a bi-directional Long Short-Term Memory (bi-LSTM) with 64 filters and 32 filters are included in the encoder network to reconstruct a clean MFCC representation, followed by a temporal Fully Connected (FC) in a decoder network. An LSTM neural network was previously used to extract speaker representation and remove noise from audio [30]–[32]. In this study, Deep-MASKS employs bi-LSTM, a variation of LSTM that considers the backward sequence in addition to the forward sequence, to learn temporal patterns over the MFCC sequence.

In the speaker matching network ($f_{\theta _2}$), the target-speaker representation vector is concatenated on every temporal slice of the speaker representation with two FC layers with 64 filters. This part of the network performs similarity matching between the speaker representation in the model and the speaker representation vector. In the last part ($f_{\theta _3}$), this latent vector is concatenated to the input MFCC followed by an LSTM with 64 filters and a temporal FC layer to reconstruct and refine the speech-separated MFCC. The size of the filters in the last FC layer must match the number of MFCC features, which we specify to be 13 in this study. Batch normalization and ReLU activation layers follow every FC layer. The final loss function is defined in 3.

\begin{equation*}
L = \sum _{i=0}^{n}\sum _{j=0}^{13}{(y_{ij}-f_{\theta _1,\theta _2,\theta _3}(x_{ij}))^2} \tag{3}
\end{equation*}

## III. Experimental Evaluation

Deep-MASKS was evaluated on a mixture of speech from multiple speakers using three reconstructions errors: Mean Squared Error (MSE), Kullback-Leibler (KL) divergence and Jensen-Shannon (JS) divergence. This section describes the preparation of the corpus and data, followed by experimental setup and baseline models.

### A. Corpora and Data Preparation

#### AMI corpus

The AMI meeting corpus [33] contains 100 hours of video and audio recordings of naturally occurring (non-scenario) as well as elicited (scenario) meetings. The dataset was originally gathered to study the effectiveness of work groups and has been utilized for various speech research such as as speaker diarization and overlapped speech detection [19], [20]. In the scenario meetings, participants were assigned and played various roles in a design team in varied contexts. The non-scenario meetings are actual meetings and speakers were not assigned to any roles or contexts. The audio was recorded using close-talking and far-field microphones with an annotation that represent speakers. This study only used audio from the AMI corpus that was recorded by a microphone on a headset and contains recordings in which approximately 18% are non-speech, 66% are speech without any overlap, and 16% are overlapped speech. The overlapped speech in this dataset is actual cross-talk, where we consider the recording from this microphone as clean speech. We simulated the dynamics of near-field talking on smartphones by using only the recordings captured by individual headsets.

Overlapped speech mixtures were generated by combining the audio from multiple headsets recorded simultaneously in the same meeting. This mixture consists of continuous recordings containing both speech and non-speech segments except for a speech utterance used as a reference for the target speaker representation vector. For evaluation purposes, the reference utterance was determined by selecting the speaker representation ($V_i$) with the highest cosine similarity, as shown in 4, to all other speech utterances ($\mathbf {V}$) in the clean speech as a speaker enrollment process.

\begin{equation*}
\mathop{\text{arg max}}\limits _{i} \frac{ V_i \cdot \mathbf {V}}{\Vert V_i \Vert \Vert \mathbf {V} \Vert } \tag{4}
\end{equation*}

We evaluated the Deep-MASKS on all possible subsets of 1-4 speakers; however, mixtures with five speakers were not evaluated as the number of overlapped segments with five speakers was considered insufficiently large.

#### Feature extraction

MFCC features were extracted using Kaldi [34] configured to compute 13 MFCCs with a window length of 25 ms and a step length of 10 ms. Kaldi is a speech processing framework built specifically for ASR tasks, which eased the integration of our speech separation method into an ASR that is included in the supplementary materials. The MFCCs were split into chunks (data instances) of 6000 MFCC frames (1 minute) to generate mini-batches during training. Each mini-batch contained 8 data instances (6000 MFCC frames, or 1 minute) randomly selected to train the Deep-MASK model.

#### Speaker representations

To implement the i-vector and x-vector, we followed the Kaldi recipes provided by [26] but modified MFCC feature extraction to match the Deep-MASK. For the d-vector speaker embedding, we followed the implementation of PyanNet [19] but extracted MFCCs to train the model with a triplet loss function instead of a waveform. All speaker representation methods were pre-trained on the whole VoxCeleb dataset [35] with slight augmentation of speech utterance in AMI training sets that were longer than 5 seconds. VoxCeleb consists of short clips of human speech, extracted from interview videos uploaded to YouTube. The resulting i-vector, x-vector, and d-vector have dimensions of 400, 512, and 512, respectively.

#### Normalization parameters

MFCCs were normalized using Short-Time Cepstral Mean and Variance Normalization (STMVN) [36] to reduce the segmental differences between speakers. We applied STMVN with a sliding window ($w$) of 3 seconds, as shown in 5 where $C(m,k)$ denotes MFCC at frame $m$ and $k^{th}$ coefficient; $\mu (m,k)$ and $\sigma (m,k)$ are mean value and standard deviation of $k^{th}$ coefficient over $w$ frames, accordingly.

\begin{align*}
C_{STMVN}(m,k) &= \frac{C(m,k)-\mu (m,k)}{\sigma (m,k)}\tag{5}
\\
\mu (m,k) &= \frac{1}{w}\sum _{i=m-0.5\,w}^{m+0.5\,w} C(i,k)\tag{6}
\\
\sigma (m,k) &= \frac{1}{w}\sum _{i=m-0.5\,w}^{m+0.5\,w} (C(i,k)-\mu (m,k))^2 \tag{7}
\end{align*}

Normalization parameters were computed from the mixture and applied to the mixture and also individually on each Mel coefficient in the ground truth MFCCs.

### B. Deep-MASKS: Speech Separation Experiment

#### Pre-training

**Fig. 4.**
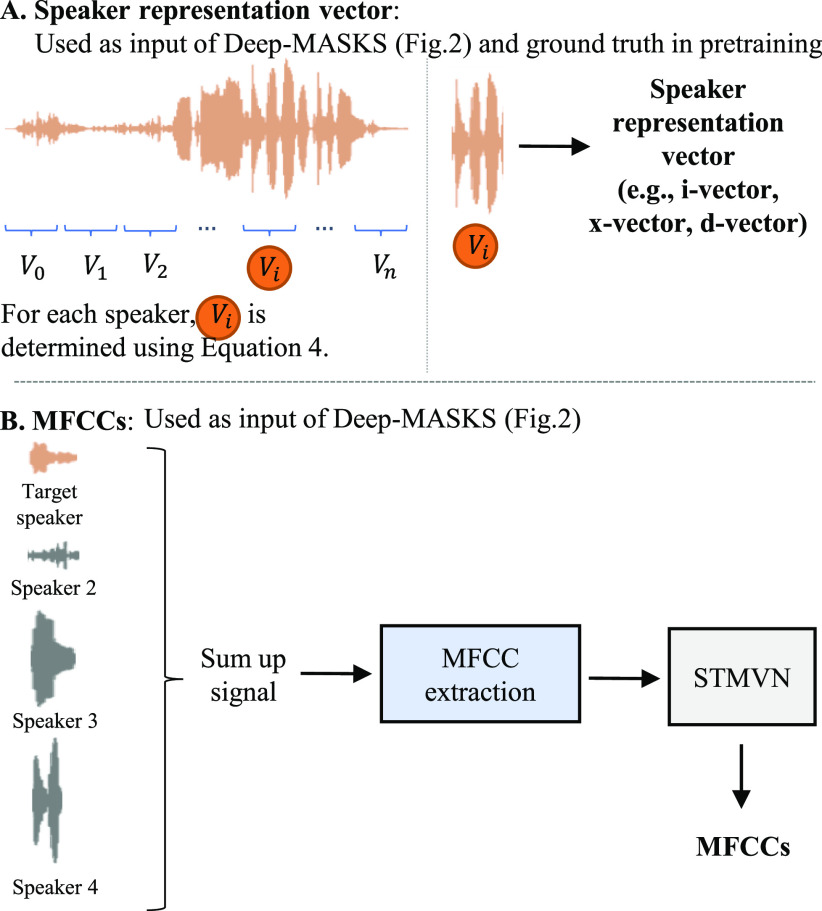
Data preparation steps for Deep-MASKS evaluation.

The encoder part of the Deep-MASKS network was trained to extract the speaker representation from MFCCs using the speaker representation vector, generated as visualized in Fig. 4(A), as one of the targets. The model was trained on each embedding method separately until the loss stopped decreasing, usually within five epochs.

#### Training

All data included the MFCC mixture, speaker representation vector, and ground truth MFCC that were pre-generated. The MFCC mixture was prepared as visualized in Fig. 4(B) while ground truth MFCC was prepared similarly but without summing across speakers. We used the Tensorflow library [37], a computation library that has implementations of state-of-the-art DNN models. We trained the proposed model on NVIDIA K80 and NVIDIA V100 GPUs with a batch size of 8, randomly selected from the data. RMSprop [38] was used to optimize the parameters based on Mean Squared Error (MSE) loss ( 3) in the mini-batch. The training was evaluated on the epoch with the lowest validation loss after training for 20 epochs.

#### Evaluation and metrics

To evaluate overlapped speech utterances, only speech segments were evaluated based on the number of speakers they contained. For continuous recording, we evaluated the model on the whole recording that contains for instance, a mixture of 3 headsets, non-speech segments, speech without overlap, two-speakers overlapped speech, and three-speaker overlapped speech.

We evaluated the reconstructed MFCC on the MSE, Kullback-Leibler (KL) divergence [39] and Jensen-Shannon (JS) divergence [40] metrics. MSE was computed using the loss function in 3 and was normalized across coefficients since the ranges of Mel coefficients are wider in lower coefficients. The KL-divergence measured the difference between the clean MFCC vs. noisy MFCC distributions, as previously used in [41], [42]. Intuitively, KL-divergence $D_{KL}(p||q)$ measures the inefficiency of using $q$ to approximate $p$. [43]. $D_{KL}(p||q)$ is computed using 8 where $p(x_i)$ and $q(x_i)$ is a normalized histogram bin $i$ of clean MFCC and mixture MFCC respectively. We quantized values in each MFCC to a histogram of 50 bins and add a constant ($1\times 10 ^{-8}$) to $p(x_i)$ and $q(x_i)$ to preserve numerical stability. If p has a uniform distribution, the Shannon entropy of distribution p is $-\log(0.02)$, which needs 5.64 bits to represent $p$. Additional bits needed to describe $p$ from $q$ ($H(p,q)$) can be computed as $H(p)+D_{KL}(p||q)$. We also used JS-divergence, another distribution measurement metric, as KL-divergence is not symmetric (i.e., $D_{KL}(p||q) \ne D_{KL}(q||p)$). JS-divergence extends KL-divergence by introducing $r$ as a mixture between $p$ and $q$, and measures the loss of using $r$ to describe $p$ and $q$. This can be computed as in 9. In our work, in addition to the signal loss captured by KL-divergence, JS-divergence also captures artifacts generated by the Deep-MASKS model.

\begin{align*}
D_{KL}(p||q)&=\sum _{i=1}^{50} p(x_i) \text{log}\frac{p(x_i)}{q(x_i)} \tag{8}
\\
D_{JS}(p||q)&=\frac{1}{2}D_{KL}(p||r) +\frac{1}{2}D_{KL}(q||r),\\
&\quad \text{ where }r=(p+q)/2 \tag{9}
\end{align*}

### C. Baseline

We compared Deep-MASKS to two state-of-the-art speech separation methods: Deep Attractor Network (DAN) [21] and Voice-Filter-Lite (VFL) [44].

We compared Deep-MASKS against other two state-of-the-art speech separation methods: Deep Attractor Network (DAN) [21] and Voice-Filter-Lite (VFL) [44].

*DAN* [21] is a speaker-independent speech separation method proposed for a mixture with an unknown number of speakers. In this method, an “attractor” is created to represent a speaker using a centroid of an embedding space. In our implementation of DAN, we specify the number of centroids to match the number of speakers in each mixture. We also computed the error of DAN, based on the speaker centroid that is closest in distance to the clean speech (lowest error), and extract MFCCs using the same configuration for the AMI dataset.

*Voice-Filter-Lite (VFL)* [44] is a speech separation method that exploits d-vector to identify target speakers. The architecture of VFL was extended from the previously proposed VoiceFilter [22], but the two-dimensional CNN layers were replaced by a one-dimensional CNN layer. We selected VFL over the VoiceFilter because the input of the VFL is a log-Mel filterbank, which is a closer speech representation to MFCCs. We reduced the number of filterbanks in VFL from 128 to 40 to make it consistent with Deep-MASKS. The architecture of the final VFL model architecture is the same that proposed by Wang *et al.* [44].

## IV. Results

**Fig. 5.**
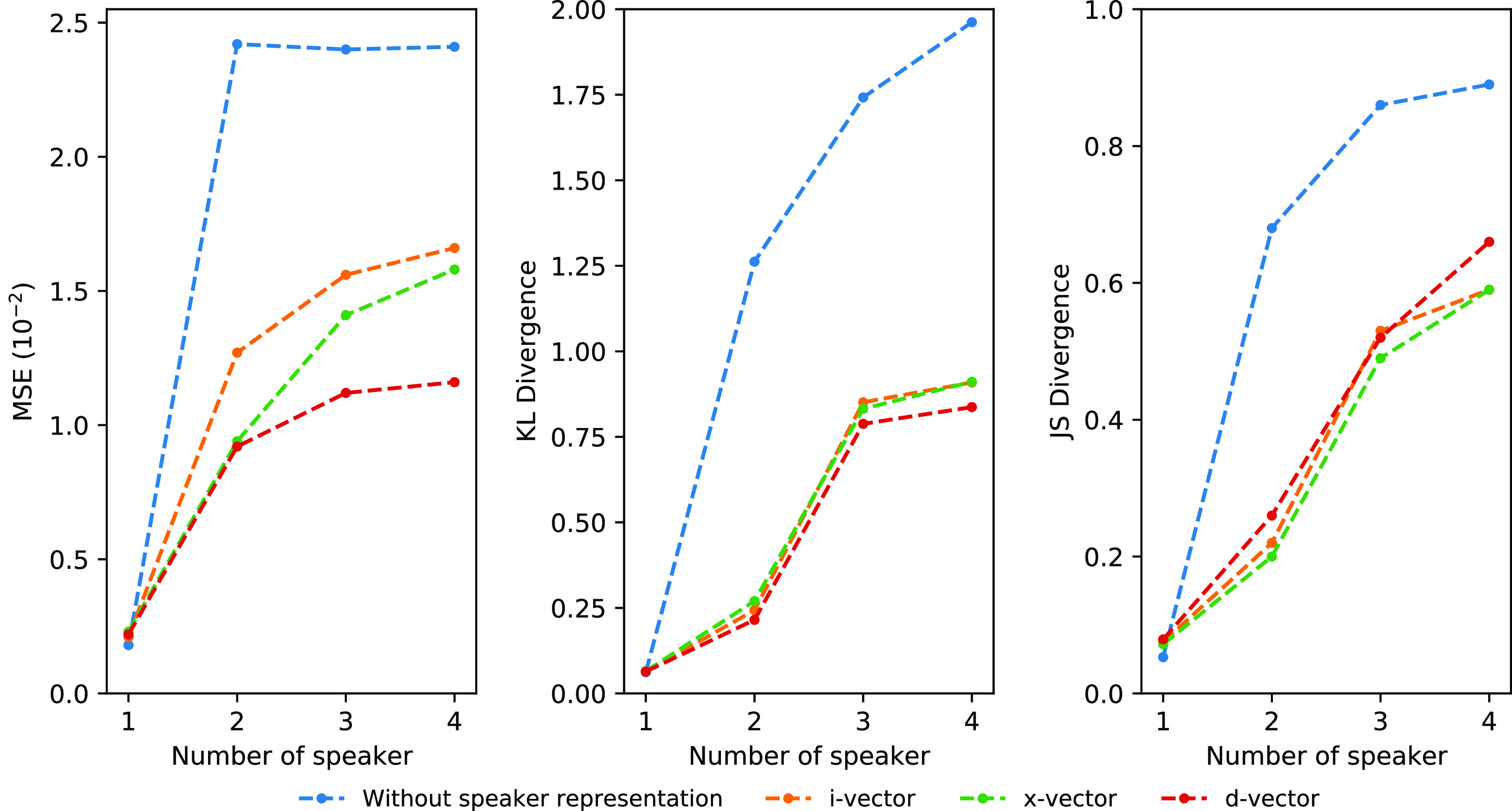
Normalized MSE, KL-divergence and JS-divergence errors of MFCC reconstructions in overlapped speech.

Tables I and II show the MSE, KL-divergence and JS-divergence errors for reconstructing MFCCs on the AMI corpus. Only overlapped speech segments are evaluated in Table I with two to four speakers. The mean and standard error (in parentheses) of all metrics are computed using 10-fold cross-validation. Using normalized MSE as the comparison metric, d-vector outperforms x-vector and i-vector in overlapped speech with more than one-speaker experiments. The differences between these speaker representation methods are more prominent on the KL-divergence plot in Fig. 5, showing that d-vector is significantly more superior to i-vector and x-vector. Compared to the two baselines, Deep-MASK with d-vector has a significantly lower normalized MSE error than VFL and DAN while slightly outperforming VFL and DAN on the KL-divergence and JS-divergence metrics. For all methods, increasing the number of speakers in the overlapped speech increased the MFCC reconstruction error as measured by all three metrics, especially going from two to three speakers.

**TABLE I:** Normalized MSE, KL-Divergence and JS-Divergence of MFCC Reconstructions in Overlapped Speech (Bold Indicates the Lowest Error for Each Speaker)

**Number**	**Deep-MASKS**				**Baselines**	
**of speaker**	**empty vector**	**i-vector**	**x-vector**	**d-vector**	**VFL**	**DAN**
**Normalized MSE** (in a unit of $10^{-2}$)					
1	0.18 (0.02)	0.21 (0.02)	0.23 (0.03)	0.22 (0.03)	0.23 (0.02)	-
2	2.42 (0.04)	1.27 (0.03)	0.94 (0.03)	**0.92 (0.04)**	0.99 (0.04)	1.63 (0.07)
3	2.40 (0.06)	1.56 (0.05)	1.41 (0.06)	**1.12 (0.06)**	1.32 (0.06)	2.26 (0.11)
4	2.41 (0.05)	1.66 (0.06)	1.58 (0.05)	**1.16 (0.05)**	1.42 (0.05)	2.22 (0.10)
**KL-divergence**					
1	0.062 (0.008)	0.065 (0.008)	0.065 (0.008)	0.064 (0.008)	0.063 (0.008)	-
2	1.26 (0.02)	0.24 (0.01)	0.27 (0.02)	**0.21 (0.01)**	0.21 (0.02)	0.37 (0.05)
3	1.74 (0.02)	0.85 (0.01)	0.83 (0.01)	**0.79 (0.02)**	0.80 (0.02)	0.91 (0.04)
4	1.96 (0.02)	0.91 (0.01)	0.91 (0.01)	**0.84 (0.01)**	0.86 (0.02)	0.95 (0.06)
**JS-divergence**					
1	**0.053 (0.006)**	0.075 (0.008)	0.072 (0.007)	0.079 (0.008)	0.081 (0.011)	-
2	0.68 (0.02)	0.22 (0.01)	**0.20 (0.01)**	0.26 (0.02)	0.29 (0.02)	0.25 (0.02)
3	0.86 (0.02)	0.53 (0.01)	**0.49 (0.01)**	0.52 (0.02)	0.54 (0.02)	0.64 (0.02)
4	0.89 (0.02)	0.59 (0.01)	**0.59 (0.01)**	0.66 (0.02)	0.67 (0.02)	0.67 (0.01)

**TABLE II:** Normalized MSE, KL-Divergence and JS-Divergence of MFCC Reconstructions in Continuous Recordings (Bold Indicates Lowest Error for Each Maximum Speaker)

**Maximum number**	**Deep-MASKS**				**Baselines**	
**of speaker**	**empty vector**	**i-vector**	**x-vector**	**d-vector**	**VFL**	**DAN**
**Normalized MSE** (in a unit of $10^{-3}$)					
1	0.845 (0.011)	0.844 (0.012)	0.849 (0.011)	**0.763 (0.011)**	0.775 (0.012)	-
2	1.178 (0.012)	1.147 (0.012)	1.075 (0.012)	**1.069 (0.013)**	1.071 (0.012)	1.152 (0.011)
3	1.184 (0.014)	1.149 (0.014)	1.075 (0.014)	**1.070 (0.014)**	1.741 (0.012)	1.155 (0.011)
4	1.183 (0.018)	1.149 (0.018)	1.075 (0.017)	**1.070 (0.018)**	1.743 (0.012)	1.155 (0.011)
**KL-divergence**					
1	0.012 (0.002)	0.012 (0.003)	0.013 (0.002)	0.012 (0.002)	0.012(0.002)	-
2	1.05 (0.03)	0.25 (0.02)	0.26 (0.03)	**0.21 (0.02)**	0.21 (0.02)	0.62 (0.03)
3	1.06 (0.03)	0.24 (0.02)	0.26 (0.03)	**0.21 (0.01)**	0.21 (0.01)	0.62 (0.03)
4	1.05 (0.03)	0.24 (0.02)	0.25 (0.03)	**0.21 (0.01)**	0.21 (0.01)	0.62 (0.03)
**JS-divergence**					
1	**0.008 (0.001)**	0.009 (0.008)	0.009 (0.007)	0.009 (0.008)	0.009 (0.008)	-
2	0.32 (0.05)	0.14 (0.03)	0.10 (0.02)	**0.08** (0.02)	0.08 (0.02)	0.18 (0.03)
3	0.32 (0.05)	0.14 (0.03)	0.10 (0.01)	**0.08** (0.02)	0.08 (0.02)	0.18 (0.03)
4	0.32 (0.05)	0.14 (0.03)	0.11 (0.02)	**0.08** (0.02)	0.08 (0.02)	0.18 (0.03)

**Fig. 6.**
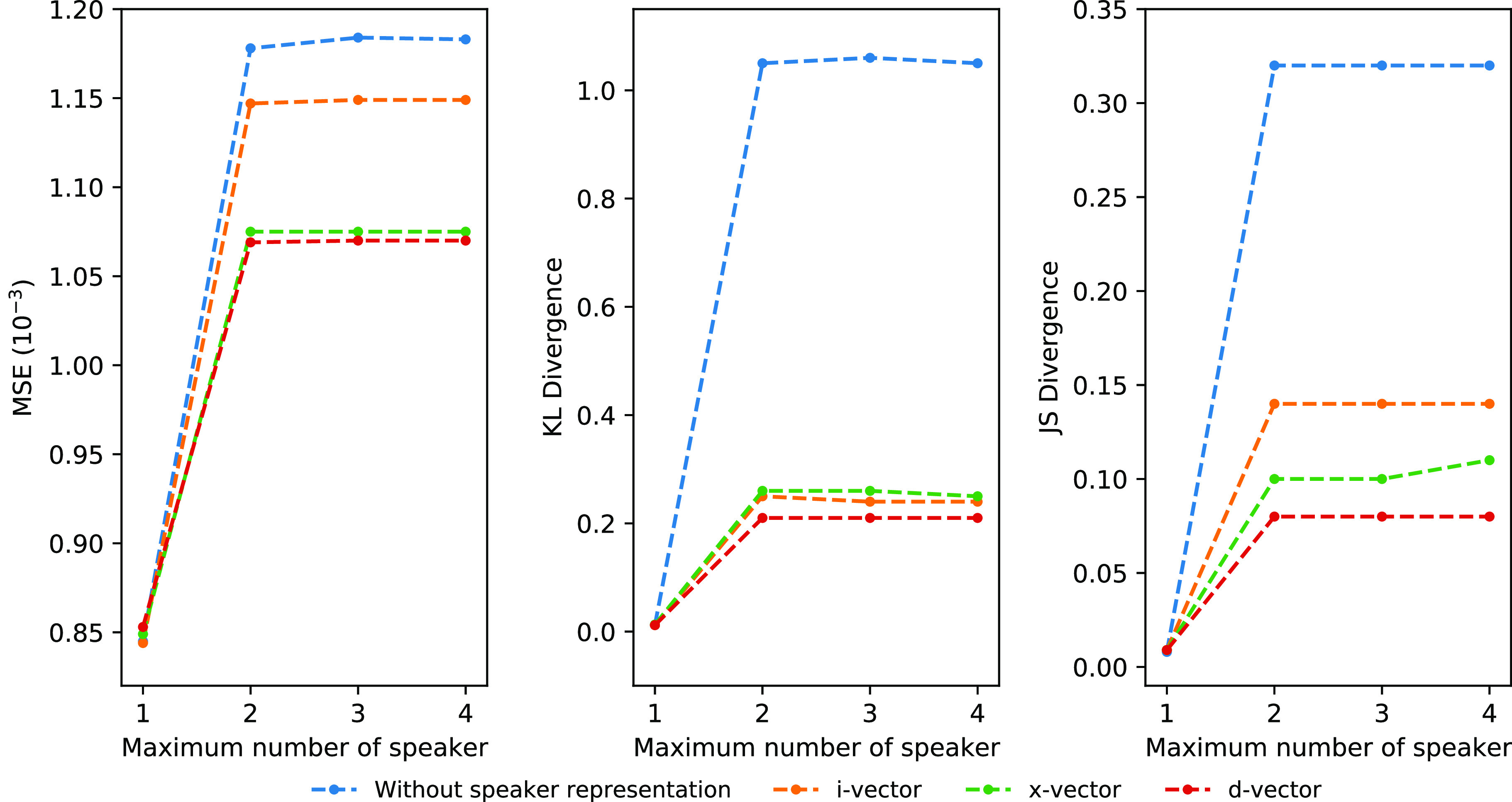
Normalized MSE, KL-divergence and JS-divergence of MFCC reconstructions in continuous recordings.

**Fig. 7.**
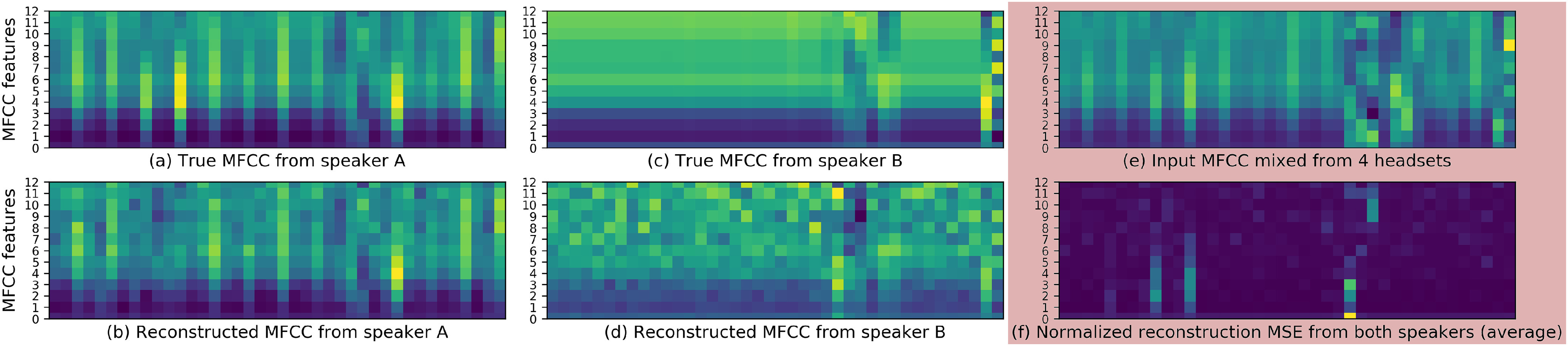
Speech-separated MFCC features reconstruction from two speakers mixture (with channel-wise normalization).

The results of continuous recording are shown in Table II and Fig. 6. The MFCC reconstruction errors in a one-speaker-maximum experiment show equal MSEs for the empty vector, i-vector, and x-vector while d-vector has a lower normalized MSE error. In scenarios with more than one speaker, d-vector has lower errors than other speaker representation methods and the two baselines on all three metrics. To assess the effectiveness of speech-separated MFCCs visually, we visualized the enhanced MFCCs of two speakers in Fig. 7. The proposed method is able to remove MFCC components that are produced by other speakers in the meeting with some minor artifacts generated.

## V. Discussion

In the experiment with no overlapped speech (one speaker), Deep-MASKS has a slightly higher MSE than the empty vector due to some artifacts generated by the networks. The presence of artifact is supported by the fact that the JS-divergences are lower than KL-divergences in an empty vector but higher when using the speaker representation method as well. Among the three speaker representations, d-vector achieves the lowest MSE for speech separation on MFCCs. However, it is worth noting that the d-vector might generate more artifacts, as reflected in a higher JS-divergence than x-vector. The performance gaps between i-vector and d-vector might be due to the use of PLDA in training the i-vector speaker representation method. In all speaker representations, the errors are increased as the number of speaker increased, especially from two to three speakers. Compared to the VFL and DAN baselines using normalized MSE, Deep-MASKS with any speaker representations outperforms the DAN method, while only Deep-MASKS with d-vector outperforms VFL. One reason that Deep-MASK outperforms VFL, although they exploit d-vector and share some similarity in their architectures, is due to the pre-training step (Section III-B). Pre-training the encoder network in Deep-MASKS allows the network to adapt to any speaker representation, which is not restricted to the D-vector as in VFL, prior to learning the speech separation. Another reason that Deep-MASK outperforms VFL is the choice of using a DNN for signal reconstruction instead of a mask. While using a mask limits the final operation to be a linear operation, Deep-MASKS utilizes a DNN approach to reconstruct the signal, yielding a more accurate, higher order function.

In the experiments with continuous recording, we note that the d-vector is more robust to non-speech segments and specifically to speech separation of MFCC that has no prior VAD applied compared to other speaker representation methods, which create some arbitrary values. KL-divergence and JS-divergence errors are consistent with the MSE, in that the maximum number of the speaker does not affect the reconstruct errors substantially when going from two to four speakers. This is likely due to the low ratio of overlapped speech frames compared to non-speech and non-overlapped frames.

Deep-MASKS is capable of mitigating the cross-talk, which is inevitable in processing speech recorded in a real-life environments, as manifested in section IV and supplementary materials, and which is a major potential hinderance to using speech in smartphone-based biomedical applications. The application of Deep-MASKS ranges from core speech technologies such as ASR, to biomedical speech assessment. Deep-MASKS ensures that the speech processed by such a system belongs to the target speaker/patient, hence improving the application’s performance and delivering accurate diagnoses. A future study will apply Deep-MASKS in passive assessment of speech impairments on smartphones.

## VI. Conclusion

We proposed Deep-MASKS, a novel speech separation method that is suited to continuous recordings as common on smartphones instead of utterances utilized in prior work. Deep-MASKS preserves speaker privacy by utilizing MFCCs and can also be adapted to a wide variety of existing speaker representations. The model uses an autoencoder to isolate the target-speaker MFCC components from overlapped speech, which improves the MFCC reconstruction error (MSE) by up to 44% compared to Deep Attractor Network that does not use speaker representations, and reduces the number of additional bits required to explain a clean MFCC from 1.22 to 0.68 using d-vector in a cross-talk scenario with two speakers. Our d-vector outperforms Voice-Filter-Lite that exploits the speaker representation for speech separation. In a comparison of the speaker representation, D-vector explicitly outperforms i-vector and x-vector in both speech utterance and continuous recording evaluations. Deep-MASKS performed especially well for overlapped speech separation, a task that is growing in importance with the rise of smartphone-based audio analyses. Future work will apply Deep-MASKS in passive assessment of speech impairments on smartphones.

## Supplementary Materials

We provide two additional experiments that evaluate Deep-MASKS for ASR and speaker diarization tasks. In the ASR, Deep-MASKS with d-vectors significantly reduces the word-error-rate by 30.87 and 49.66 in cross-talks with two and three speakers, respectively. By extending Deep-MASKS for speaker diarization, it obtains a lower diarization error rate than a baseline that performs on the cross-talk.

Supplementary Materials
