# Supplementary material for: Privacy-Preserving Deep Speaker Separation for Smartphone-Based Passive Speech Assessment
Source: IEEE Open J Eng Med Biol. 2021 Mar 4;2:304–13. doi: 10.1109/OJEMB.2021.3063994 (PMC8940203; doi:10.1109/OJEMB.2021.3063994)
Supplement: Supplementary Materials [file supp1-3063994.pdf]

Supplementary Materials

Privacy-preserving Mel-Frequency Cepstrum Coefficients (MFCC) based Speech Separation for Smartphones using Speaker Representations and an Autoencoder

Apiwat Ditthapron, Emmanuel O. Agu, and Adam C. Lammert

THE first section of supplementary materials describes steps in Mel-Frequency Cepstrum Coefficients (MFCC) extraction process that is used in our proposed deep speaker separation based on MFCCs (Deep-MASKS). In additional to the speech separation results presented in the main manuscript, we demonstrate the effectiveness of Deep-MASKS for Automatic Speech Recognition (ASR), in Section II, and speaker diarization, in Section III.

I. Mel-Frequency Cepstrum Coefficients (MFCCs)

MFCC is a short-term audio feature that has been overwhelmingly utilized in traditional speech recognition systems and recently used in speech assessment applications [1]–[3]. With the current computing power of smartphones, it is feasible to perform MFCC extraction on smartphones and preserve privacy information to some extent. Steps in MFCC extraction are as follows.

1) Signal pre-processing and framing: The signal processing method varies across the applications. However, it is common to apply a pre-emphasis filter to boost high frequency and apply a bandpass filter to remove some noise. The pre-emphasized signal (s') at time t is computed using the Equation 1, where s(t) is a raw audio signal at time t and α is a coefficient, usually, between 0.90 and 0.97. In speech processing, it is common to limit the maximum frequency to about the Nyquist frequency. [4] suggests extracting MFCC features between 40 Hz and 7800 Hz for audio signals with a sampling rate of 16000 Hz.
$$s'(t) = s(t) - \alpha s(t-1) \quad (1)$$
Then, the audio is split into a small frame of 20-40 ms with 10 ms. Subsequent extraction steps are performed on a short duration of frame audio signal.
2) Windowing and Discrete Fourier Transform (DFT): To smooth and prevent discontinuities in the signal, particularly, at the beginning and end of each frame, hamming window w_{ham}, as shown in Equation 3 is applied on the time domain signal ($s_i(n)$) during DFT, Equation 2, where N denotes frame size. The DFT transforms the signal at frame i (s_i) to a complex DFT (S_i) of DCT window size K. Then, power spectrum is obtained by Equation 4.
$$S_i(k) = \sum_{n=1}^{N} s_i(n) w_{\text{ham}}(n) e^{-j\frac{2\pi}{N}nk}, 1 \le k \le K \quad (2)$$
$$w_{\text{ham}}(n) = 0.54 - 0.46cos(\frac{2\pi n}{N-1}), 0 \le n \le N-1. \quad (3)$$
$$P_i(k) = \frac{|S_i(k)|^2}{N} \quad (4)$$
3) Mel Filterbank: Mel-filter banks mimic humans' sound perception that is non-linear and more discriminative at a lower frequency than higher frequency. Thus, magnitude coefficients are passed through triangular filter, accumulating weighted magnitude into filterbank spectral magnitude. M filters bank (H_m) can be computed as in the Equation 5-7.
$$m(f) = 2595\log_{10}(1+\frac{f}{700}) \quad (5)$$
$$f(m) = 700(10^{m/2595} - 1) \quad (6)$$
$$H_m(k) = \begin{cases} 0 & k < f(m-1) \\ \frac{k-f(m-1)}{f(m)-f(m-1)} & f(m-1) \le k \le f(m) \\ \frac{f(m+1)-k}{f(m+1)-f(m)} & f(m) \le k \le f(m+1) \\ 0 & k > f(m+1) \end{cases} \quad (7)$$
4) Logarithm and inverse DFT: After applying Mel-filterbanks on the periodogram, base-10 logarithm is applied and followed by a Discrete Cosine Transform (DCT) to reduce the high correlation between each filterbank and results in the form of Mel-frequency cepstral coefficients. In most applications, coefficients higher than 14 are discarded.
5) Differential and acceleration MFCCs Deltas and Delta-Deltas MFCCs are often used to boost the ASR performance by providing the dynamic information of MFCCs over time in additional to the MFCCs. Each delta MFCC at frame t (d_t) can be computed as in Equation 8, where N is a window size; c_t is MFCC at frame t. Delta-Deltas MFCCs computation is the same as Deltas MFCCs, but Delta MFCC is used instead of static MFCC.
$$d_t = \frac{\sum_{n=1}^{N} n(c_{t+n} - c_{t-n})}{2\sum_{n=1}^{N} n^2} \quad (8)$$

While MFCCs are widely used to preserve speaker privacy, prior work has demonstrated that substantial intelligible information can be reconstructed from MFCC features [5], [6]. Milner *et al* demonstrated that speaker-dependent reconstruction is more audible than the corresponding speaker-independent reconstruction [5]. Juvela *et al* [6] shows that Generative Adversarial Networks (GANs) can synthesize high quality, realistic audio. Parthasarathi *et al* [7] proposed a Linear Prediction (LP) residual of real-cepstral and Mel-Frequency Cepstral

Coefficients (MFCC) methods to create privacy-sensitive audio features for speaker diarization. However, to the best of our knowledge, these privacy-preserving features have not yet been evaluated for speech assessment and speaker diarization tasks. Thus, we continue to adopt MFCC features in this work as they maintain speaker privacy better than raw audio and are still widely adopted for speech assessment. Moreover, [8], [9] demonstrated that MFCCs can be collected remotely and analyzed on a local server and ensure a high voice-privacy level using encryption algorithms designed specially for MFCCs.

II. Automatic Speech Recognition (ASR)

As an objective measurement of Deep-MASKS, we evaluated its speech separation in a well-known ASR system.

Methodology: The Deep-MASKS network was incorporated into the Kaldi S5 ASR system [4]. The S5 ASR system is an MFCC-based ASR training recipe on the Kaldi framework with components and hyperparameters tuned specifically for the Librispeech corpus. We replaced the MFCC extraction step in the S5 recipe with Deep-MASKS trained on the Librispeech corpus with a modification of MFCC components to include delta and 2nd delta MFCC. We also used three-speaker representations in place of the i-vector that is used in the S5 recipe. sMBR, a time delay neural network with sequence training [10] was applied on MFCC features to learn long-term temporal dependencies from short-term speech features. The experiment was performed on a train-clean set and evaluated on a test-clean set using an fglarge decoding graph. The decoding graph is a language model containing probabilities of word sequences. We adopted the fglarge decoding graph that was trained for a sequence of 4 words (4-gram) without pruning as has been previously used in [10].

Evaluation and metric: We evaluated the effect of applying Deep-MASKS in a well-known ASR system using Word Error Rate (WER), as described in Equation 9. Librispeech mixtures of two to four speakers were used to manifest this efficacy.

$$\mathrm{WER} = \frac{\text{substitution words} + \text{insertion words} + \text{deletion}}{\text{reference words}} \tag{9}$$

Baseline: [11] evaluated their proposed speech separation, called Voice-Filter-Lite (VFL), for the ASR task but using a different ASR system from ours. We replicated the VFL as explained in the main manuscript to obtain speech separated MFCCs. We compared our proposed method against VFL using WER.

Results: To further evaluate our proposed method, we examined the performance gained from fusing Deep-MASKS in the ASR pipeline. We compared our proposed Deep-MASKS against VFL [11] as shown in Table I. In the speech mixture with two speakers, the ASR achieves WERs of 12.48% and 63.63% on the clean speech and overlapped speech respectively without applying any speech separations. Clean WER reflects a degradation from applying speech separation on a clean speech (no cross-talk), and overlapped WER measures the ASR performance on the speech with more than one speaker. Based on the improvement percentage, that is relatively computed on the overlapped WER, Deep-MASKS with d-vector has a higher improvement percentage than the other two speaker representations, and outperforms VFL baseline by 2.83. Similarly in the three-speakers scenario, d-vector performs better than the other speaker representations and outperforms VFL by 4.30 on the improvement percentage metric. Although this experiment is not conducted on the speech assessment, which is our ultimate objective, it shows promising results in coping with the cross-talk problem that degrades ASR and speech assessments.

III. Speaker diarization

The speaker diarization system in the traditional pipeline might gain access to only a small amount of data in recordings with heavy cross-talks. In this experiment, we show that Deep-MASKS can be extended for speaker diarization that considers all overlapped speeches.

Methodology: We first applied Deep-MASKS on continuous MFCCs to remove cross-talk. VAD Bredin2020 was trained to predict voice activity annotations for each speaker. Thereafter, annotations from all speakers in the same meeting were combined to create speaker diarization results. We did not remove the enrollment period from the dataset for comparison purposes.

Evaluation and metric: Our speaker diarization evaluation is similar to that in the Pyannote library Bredin2020, which is a DNN-based speaker diarization module and contains the evaluation pipeline for the AMI dataset. Training sets and development sets were used during the VAD training process. We report Diarization Error Rate (DER), which is expressed as the following equation.

$$DER = E_{\text{false alarm}} + E_{\text{missed detection}} + E_{\text{speaker confusion}} \tag{10}$$

Baseline: Overlap-aware speaker diarization using overlapped speech detection Bullock2020 and Pyannote speaker diarizationBredin2020 were used as baselines trained using the AMI dataset. The VAD and speaker embedding components were trained on the VoxCeleb dataset nagrani2017voxceleb. The VoxCeleb dataset contains short utterances from more than 1,000 celebrities, collected from YouTube videos.

Result: We extended Deep-MASKS for speaker diarization by applying an MFCC-based VAD on the separated speech and evaluated them on the AMI corpus. The DERs of the Deep-MASKS with three speaker representations with two baselines are shown in Table II. When utilized with d-vector, the proposed method is slightly better than i-vector and x-vector. Our D-vector outperforms the other speaker representation methods and baselines with a DER of 23.4 where x-vector and i-vector are competitive with overlap-aware [12] that is implemented for speaker diarization with overlapped speech. All proposed methods are better than a speaker diarization baseline without speech separation.

References

[1] M. Wiśniewski, W. Kuniszyk-Jóźkowiak, E. Smołka, and W. Suszyński, "Automatic detection of disorders in a continuous speech with the hidden markov models approach," in *Computer Recognition Systems 2*. Springer, 2007, pp. 445–453.

[2] O. C. Ai, M. Hariharan, S. Yaacob, and L. S. Chee, "Classification of speech dysfluencies with mfcc and lpcc features," *Expert Systems with Applications*, vol. 39, no. 2, pp. 2157–2165, 2012.

TABLE I. Automatic speaker recognition with speech separation WER on Librispeech corpus

Method	Clean WER(%)	Overlapped WER(%)	Improvement percentage †
**Two speakers**			
Without speech separation	**12.48**	63.63	-
Deep-MASKS			
i-vector	13.35	41.64	34.56
x-vector	13.42	43.08	32.30
d-vector	13.25	**32.76**	**48.51**
Voice-Filter-Lite (VFL)	13.18	34.54	45.71
**Three speakers**			
Without speech separation	**12.48**	88.85	-
Deep-MASKS			
i-vector	17.62	54.36	38.81
x-vector	18.19	54.10	39.11
d-vector	18.67	**44.73**	**49.66**
Voice-Filter-Lite (VFL)	18.22	48.54	45.36

† The improvement percentage measures how much WER is improved from applying speech separations on the overlapped speech, which is computed relatively to the overlapped WER without applying any speech separation.

TABLE II. An objective measurement of speech-separated MFCC: a speaker diarization evaluation (bold indicates lowest DER)

Method	DER
Deep-MASKS	
with i-vector	24.2
with x-vector	23.8
with d-vector	**23.4**
Baseline	
Overlap-aware [12]	23.8
Without speech separation	33.6

[3] Z. Huang, J. Epps, D. Joachim, and M. Chen, “Depression detection from short utterances via diverse smartphones in natural environmental conditions.” in *Proc. Interspeech*, 2018, pp. 3393–3397.

[4] D. Povey, A. Ghoshal, G. Boulianne, L. Burget, O. Glembek, N. Goel, M. Hannemann, P. Motlicek, Y. Qian, P. Schwarz *et al.*, “The kaldi speech recognition toolkit,” in *IEEE 2011 workshop on automatic speech recognition and understanding*, no. CONF. IEEE Signal Processing Society, 2011.

[5] B. Milner and X. Shao, “Prediction of fundamental frequency and voicing from mel-frequency cepstral coefficients for unconstrained speech reconstruction,” *IEEE transactions on audio, speech, and language processing*, vol. 15, no. 1, pp. 24–33, 2006.

[6] L. Juvela, B. Bollepalli, X. Wang, H. Kameoka, M. Airaksinen, J. Yamagishi, and P. Alku, “Speech waveform synthesis from mfcc sequences with generative adversarial networks,” in *Proc. ICASSP*. IEEE, 2018, pp. 5679–5683.

[7] S. H. K. Parthasarathi, H. Bourlard, and D. Gatica-Perez, “Wordless sounds: robust speaker diarization using privacy-preserving audio representations,” *IEEE transactions on audio, speech, and language processing*, vol. 21, no. 1, pp. 85–98, 2012.

[8] M. Hadian, T. Altuwaiyan, X. Liang, and W. Li, “Privacy-preserving voice-based search over mhealth data,” *Smart Health*, vol. 12, pp. 24–34, 2019.

[9] M. Thenmozhi and K. Narmadha, “Privacy-enhanced emotion recognition approach for remote health advisory system,” in *Artificial Intelligence and Evolutionary Computations in Engineering Systems*. Springer, 2020, pp. 133–142.

[10] V. Peddinti, D. Povey, and S. Khudanpur, “A time delay neural network architecture for efficient modeling of long temporal contexts,” in *Sixteenth Annual Conference of the International Speech Communication Association*, 2015.

[11] Q. Wang, I. L. Moreno, M. Saglam, K. Wilson, A. Chiao, R. Liu, Y. He, W. Li, J. Pelecanos, M. Nika *et al.*, “Voicefilter-lite: Streaming targeted voice separation for on-device speech recognition,” *Proc. Interspeech 2020*, pp. 2677–2681, 2020.

[12] L. Bullock, H. Bredin, and L. P. Garcia-Perera, “Overlap-aware diarization: resegmentation using neural end-to-end overlapped speech detection,” in *Proc. ICASSP*, Barcelona, Spain, May 2020.
